# Light-controlled flavonoid biosynthesis in fruits

**DOI:** 10.3389/fpls.2014.00534

**Published:** 2014-10-09

**Authors:** Laura Zoratti, Katja Karppinen, Ana Luengo Escobar, Hely Häggman, Laura Jaakola

**Affiliations:** ^1^Department of Biology, University of OuluOulu, Finland; ^2^Programa de Doctorado en Ciencias de Recursos Naturales, Universidad de la FronteraTemuco, Chile; ^3^Climate laboratory Holt, Department of Arctic and Marine Biology, UiT The Arctic University of NorwayTromsø, Norway; ^4^Norwegian Institute for Agricultural and Environmental Research, Bioforsk Nord HoltTromsø, Norway

**Keywords:** anthocyanins, berries, flavonols, fruits, light, MYBs, proanthocyanidins, UV

## Abstract

Light is one of the most important environmental factors affecting flavonoid biosynthesis in plants. The absolute dependency of light to the plant development has driven evolvement of sophisticated mechanisms to sense and transduce multiple aspects of the light signal. Light effects can be categorized in photoperiod (duration), intensity (quantity), direction and quality (wavelength) including UV-light. Recently, new information has been achieved on the regulation of light-controlled flavonoid biosynthesis in fruits, in which flavonoids have a major contribution on quality. This review focuses on the effects of the different light conditions on the control of flavonoid biosynthesis in fruit producing plants. An overview of the currently known mechanisms of the light-controlled flavonoid accumulation is provided. R2R3 MYB transcription factors are known to regulate by differential expression the biosynthesis of distinct flavonoids in response to specific light wavelengths. Despite recent advances, many gaps remain to be understood in the mechanisms of the transduction pathway of light-controlled flavonoid biosynthesis. A better knowledge on these regulatory mechanisms is likely to be useful for breeding programs aiming to modify fruit flavonoid pattern.

## Introduction

Phenolic compounds constitute one of the most important groups of the bioactive compounds in food plants. These compounds possess diverse roles in signaling and defense in plants. In fruits and berries, flavonoids and hydroxycinnamic acids are the major phenolic compounds. Flavonoids are important determinants of quality and economic value of fruits as they have effect on color, aroma, astringency and antioxidant properties (He and Giusti, 2010). For example in grape berries (*Vitis vinifera*), flavonoid composition has effects on taste and quality of wine as well as conservation. Over 10,000 naturally occurring flavonoids have been described so far (Martens et al., 2010). The major flavonoid compounds present in flowers and fruits belong to flavonols, anthocyanins, and proanthocyanidins (PAs). Anthocyanin pigments are primary determinant of plant colors and serve as visual signals for pollinators in flowers and seed dispersers in ripe fruits. Flavonols have a role in photoprotection and they are generally considered to act as ultraviolet (UV) protectants and free-radical scavengers. PAs as astringent compounds can offer protection during the early stages of fruit development against herbivory and pathogen attack (Koes et al., 2005; Bogs et al., 2007).

Flavonoids are biosynthesized via the phenylpropanoid pathway and the key enzymes leading to different intermediates and different flavonoid classes are well known. At a molecular level, the biosynthesis of flavonoids is regulated via coordinated transcriptional control of the structural enzymes in the biosynthetic pathway by DNA binding R2R3 MYB transcription factors and, in many cases, interaction with MYC-like basic helix-loop-helix (bHLH) and WD40-repeat proteins (Hichri et al., 2011; Jaakola, 2013). Recent studies have revealed new upstream regulators of the pathway. In fruits, links between the key regulators of fruit development, SQUAMOSA- and SEPALLATA-class MADS box transcription factors, and anthocyanin biosynthesis have been shown in bilberry (*Vaccinium myrtillus*) and pear (*Pyrus communis*) (Jaakola et al., 2010; Wu et al., 2013).

The genetic background of the plant is the main determinant of the content of phenolic compounds in plant tissues, whereas external factors can cause qualitative or quantitative changes in the composition of these compounds. In many fruits, flavonols and PAs are the main flavonoids at the beginning of the fruit development and the accumulation of anthocyanin pigments is often an indicator of ripening. Fruits can be categorized into those which accumulate anthocyanins both in their skin and flesh, those which accumulate anthocyanins only in skin and those that accumulate anthocyanins in their skin only as response to light stimulus. In the first two classes, the developmental regulation of anthocyanin biosynthesis has a crucial role (Jaakola, 2013).

In climacteric fruits, the burst of plant hormone ethylene initiates the ripening process. In non-climateric fruits, such as grapevine, strawberry (*Fragaria* × *ananassa*), blueberry and bilberry (*Vaccinium* spp.), the plant hormone abscisic acid (ABA) seems to have a regulatory role both in ripening and initiation of anthocyanin biosynthesis (Wheeler et al., 2009; Jia et al., 2011; Zifkin et al., 2012; Karppinen et al., 2013). In addition to hormonal regulation, several studies have pointed that external factors, including temperature, light conditions, nutritional status and biotic stresses play a significant role in the accumulation of flavonoids in fruits (Koes et al., 2005; Jaakola and Hohtola, 2010; Azuma et al., 2012).

Several recent reviews have been dealing with the regulation of flavonoid biosynthesis in plants (e.g., Hichri et al., 2011; Falcone Ferreyra et al., 2012; Li, 2014). Different aspects of plant response to light have also been reviewed recently (e.g., Carvalho et al., 2011; Karpinski et al., 2013; Ballaré, 2014). In the present review, we focus on the role of light in the regulation of flavonoid biosynthesis in fruits.

## Properties of light and solar radiation

The solar irradiation reaching the earth's surface changes during the day and along the year. It is at highest around noontime, and shows higher peak between summer solstice and equinoxes, which coincides with the fruit ripening period of most of plant species. Photoperiod is not the same all over the globe as day length varies with the latitude. In northern areas, above 66°N latitude, the latitude of Murmansk-Russia, Rovaniemi-Finland, and Selawik Lake-Alaska, the sun remains continuously above the horizon in the summer, whereas at lower latitudes, for example 45°N, the latitude of Milan-Italy, Ottawa-Canada and Queenstown-New Zealand, sun shines 16 h in the longest days of the year.

**Figure 1:**
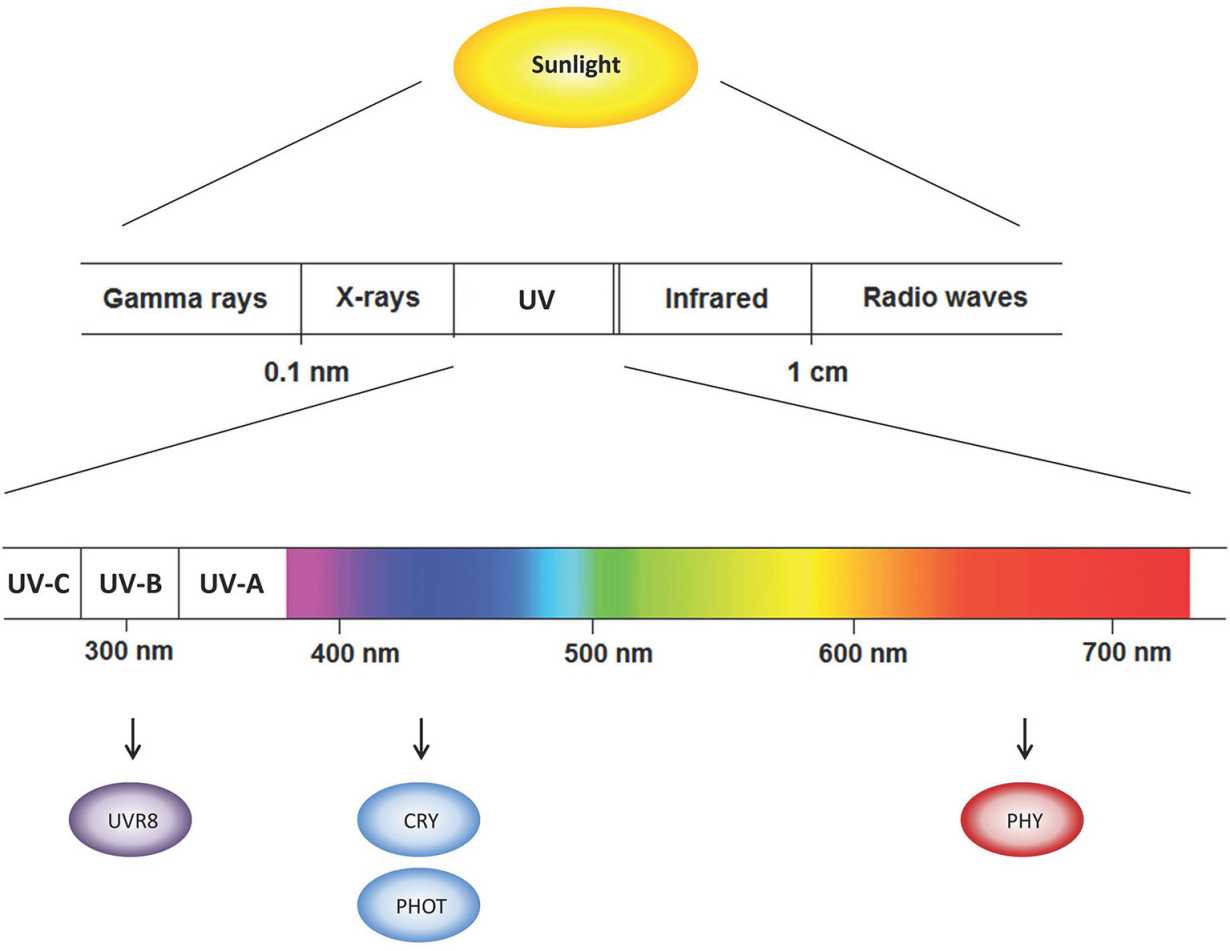
**The spectrum of solar radiation reaching from gamma rays to radio waves with closer view on visible wavelengths and plant photoreceptors absorbing specific wavelength regions**. Cry, cryptochromes; Phy, phytochromes; Phot, phototropins; UV, ultraviolet; UVR8, UV-B photoreceptor.

**Figure 2:**
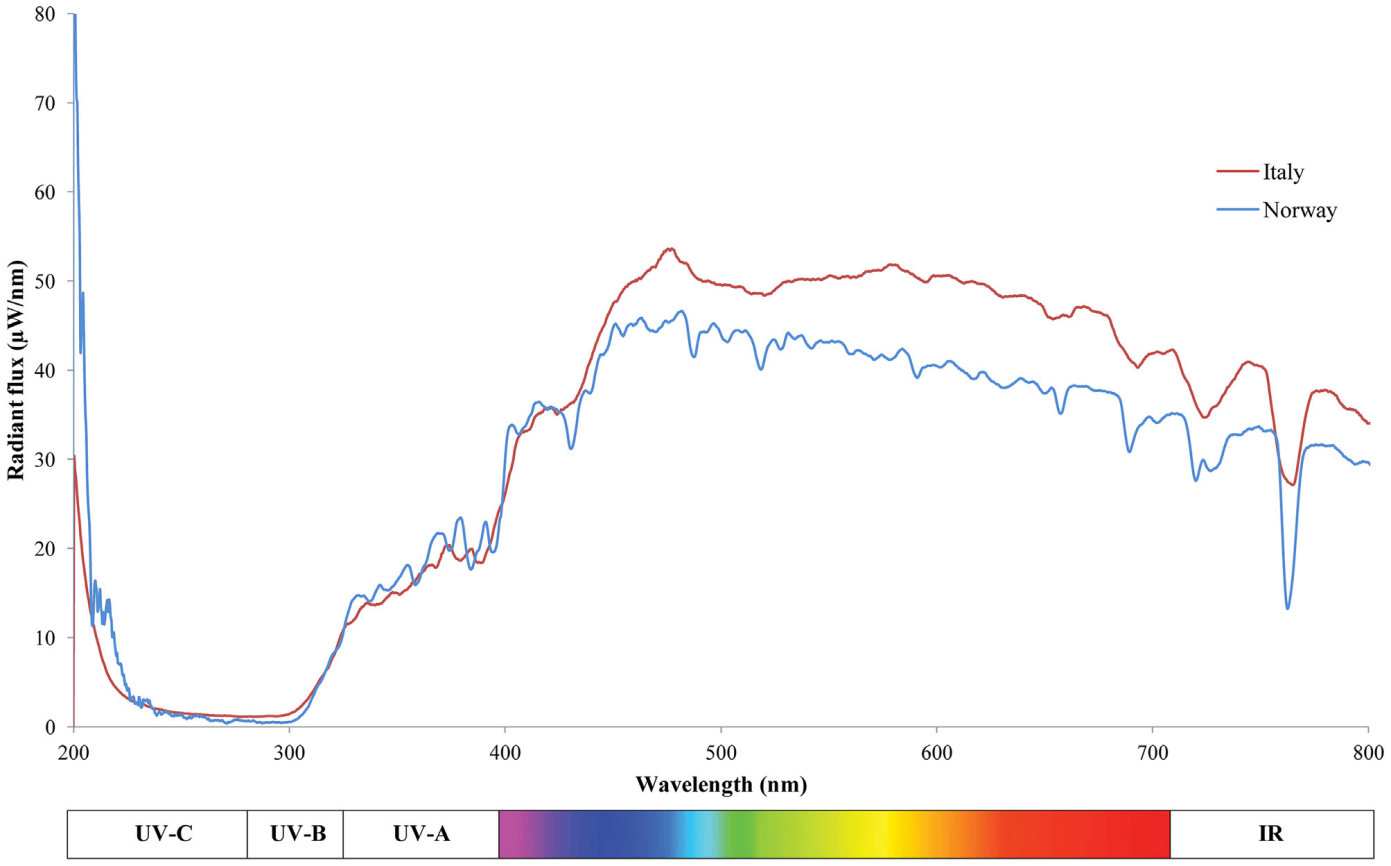
**Summer solar radiant flux spectra of two European locations (Tromsø, Norway, latitude 69°N, longitude 18°E; Trento, Italy, latitude 46°N, longitude 11°E) under clear sky and midday conditions**. Tromsø UV-B_(280–320 nm)_: 0.71 μjoule cm^−^; UV-A_(320–400 nm)_: 12.37 μjoule cm^−2^: PAR_(400–700 nm)_: 1389 μmoles photones m^−2^ s^−1^. Trento UV-B_(280–320 nm)_: 2.29 μjoule cm^−2^; UV-A_(320–400 nm)_: 45.5 μjoule cm^−2^: PAR_(400–700 nm)_: 3670 μmoles photones m^−2^ s^−1^. IR, infrared; PAR, photosynthetic active radiation.

The electromagnetic spectrum of solar radiation stretches from gamma and X-rays at one extreme to radio waves at the other (Figure 1). The biologically active radiation consists of the spectrum from approximately 300 to 800 nm including UV-light (below 400 nm). Visible light spectrum lays in the range between 400 and 710 nm and is subdivided in blue (400–495 nm), green (495–570 nm), yellow (570–590 nm), and red (590–710 nm) wavelengths. At the extreme end of the visible spectrum, is far-red (710–750 nm) light, followed by the infrared radiation. The spectrum changes daily in terms of radiation intensity, while light quality is more stable. For instance, in a location nearby Trento (Italy, latitude 46°N), our measures show that the relative amount of blue, red and far-red light reaching the earth's surface was constant throughout the day (between 9 am and 6 pm, during two consecutive weeks in June 2013). The measured visible light spectrum was composed by 17% of blue, 44% of green, 30% of red, and 9% of far-red light (Figure 2). This information is consistent with the earlier reports on the quality of the sun spectra (Robertson, 1966).

The quality of daily spectrum may vary, however, with the latitude as seen in our measurements (Figure 2). In the Arctic (latitude 69°N), Taulavuori et al. (2010) recorded an increase of the relative amount of blue and far-red light components during the “night hours” on summer solstice, while the relative proportion of red light decreased. In the Southern hemisphere, for instance New Zealand (latitude 45°S) receives 40% higher levels of UV radiation compared to similar latitudes in the Northern hemisphere (McKenzie et al., 2006; Gregan et al., 2012), and in Southern Chile (at latitude 39°S), and Australia (latitude 38°S) the received UV radiation can be even higher (Huovinen et al., 2006).

## Perception of light by plants

The ability to perceive and transduce light signal is important for optimal growth and development of sessile plants. Plants are reliant on sunlight as their source of energy and they are able to sense the different aspects of light in their growth environment including light intensity, direction, specific wavelengths and photoperiod. Plants employ a complex array of photoreceptors to coordinate their response to the ambient light environment (e.g., Wagner et al., 2005). In addition to chlorophylls and carotenoids in light-harvesting complexes participating in photosynthesis, higher plants utilize multiple sensory photoreceptors to accurately perceive light conditions ranging from UV-B to far-red wavelengths (Möglich et al., 2010; Rizzini et al., 2011). Principal among these is the phytochrome superfamily including photoreceptors absorbing red/far-red light (PHYA, PHYB, PHYC, PHYD, PHYE) as well as cryptochromes (CRY1, CRY2, CRY3), and phototropins (PHOT1, PHOT2) sensing UV-A/blue light, and UV-B photoreceptor UV RESISTENCE LOCUS8 (UVR8) that has been recently identified (Favory et al., 2009; Rizzini et al., 2011; Casal, 2013) (Figure 1). Upon light absorption, these photoreceptors activate various signal transduction cascades to regulate light-dependent responses and related gene expression in plants.

For light sensing and signaling, phytochromes consist of a bilin chromophore bonded to the protein moiety. In flowering plants, LONG HYPOCOTYL 2 (HY2) is the only ferredoxin-dependent bilin reductase (FDBR) producing the phytochromobilin for phytochromes. It has been shown that mutations in *HY2* gene cause the loss of all photoactive phytochromes in plants and furthermore lead to disruption in photomorphogenesis (Kohchi et al., 2001; Chen et al., 2012). Phytochromes exist in two different interconvertible forms; P_*r*_ that absorbs red light and far-red light absorbing P_fr_. Phytocromes are synthesized in the dark in the P_r_ form and following the conversion to the P_fr_ form, they move to the nucleus. Red light (660 nm) causes conversation of P_r_ to biologically active P_fr_ form and far-red light (730 nm) the conversation back to P_r_ form. P_fr_ can also be degraded in the proteasome after ubiquitination if not back-reverted to P_r_. Under white light containing both red and far-red wavelengths, photoequilibrium is established after few minutes. The different forms allow phytochrome to function as a biological switch, turning responses on and off enabling the detection of circadian rhythms and seasonal changes in light conditions (Reed, 1999; Casal, 2013).

Cryptochromes are flavin-containing photoreceptors for blue, green and UV-A light perception. Cryptochromes are involved in sensing circadian rhythms and regulation of many developmental and adaptive processes including biosynthesis of secondary metabolites, such as flavonoids (Giliberto et al., 2005; Lopez et al., 2012). Also blue and UV-A light absorbing phototropins have been shown to be involved in phototropism and blue light-induced chloroplast migration and stomatal opening (Briggs and Christie, 2002). Recent findings have shown that phototropins also play a role in blue light-mediated changes in biosynthesis of secondary metabolites (Kadomura-Ishikawa et al., 2013).

## Effect of light intensity on flavonoid biosynthesis in fruits

Numerous fruit bagging and shading experiments have shown the importance of light conditions on the biosynthesis and accumulation of flavonoids. Exclusion of fruits from sunlight has in many cases been demonstrated to lead suppressed expression of flavonoid pathway genes resulting in decreased amounts of flavonoid compounds in both climacteric and non-climacteric fruits. For example in pericarp of non-climacteric litchi (*Litchi chinensis*), fruit bagging treatments have been shown to inhibit accumulation of anthocyanins as well as expression of anthocyanin biosynthetic genes chalcone synthase (*LcCHS*), chalcone isomerase (*LcCHI*), flavanone 3-hydroxylase (*LcF3H*), dihydroflavonol 4-reductase (*LcDFR*), anthocyanidin synthase (*LcANS*), and UDP-glucose: flavonoid 3-*O*-glucosyltransferase (*LcUFGT*) that were again up-regulated accompanied by elevated anthocyanin accumulation after debagging and exposure the fruits to sunlight (Wei et al., 2011).

Extensive studies have been carried out on the effect of light conditions on fruit flavonoid composition in non-climacteric grapevine fruits. The studies demonstrate that grape berries adapt to high light by elevating the expression of an array of both early and late flavonoid biosynthetic genes in berry skin which leads in the increased content of anthocyanins, PAs as well as flavonols (Jeong et al., 2004; Cortell and Kennedy, 2006; Fujita et al., 2006; Pereira et al., 2006; Matus et al., 2009; Azuma et al., 2012; Koyama et al., 2012). In the study of Azuma et al. (2012), light treatment led to significantly higher total anthocyanin content in grape berry skin compared to dark grown berries, and induced higher expression levels of *CHS, CHI, F3H*, flavonoid 3′,5′-hydroxylase (*F3*′*5*′*H*), DFR, *O*-methyltransferase (*OMT*) as well as *UFGT*. Exposure to light was shown to increase anthocyanin concentrations in grape berry skin regardless of ambient temperature (Spayd et al., 2002; Azuma et al., 2012). Especially flavonol levels seem to be sensitive to changes in light conditions in grape berries, as highly induced accumulation of flavonols along with increased expression of flavonol synthase (*FLS*), has been reported by light exposure in different cultivars such as Shiraz (Downey et al., 2004), Merlot (Fujita et al., 2006), Cabernet Sauvignon (Fujita et al., 2006; Matus et al., 2009; Koyama et al., 2012) as well as Pione (*Vitis* × *labruscana*, Azuma et al., 2012).

Positive effects of light on flavonoid biosynthesis has also been reported in many other fruit species including Chinese bayberry (*Myrica rubra*, Niu et al., 2010), cranberry (*Vaccinium macrocarpon*, Zhou and Singh, 2004), bilberry (Uleberg et al., 2012), raspberry (*Rubus idaeus*, Wang et al., 2009a), and tomato (*Solanum lycopersicum*, Løvdal et al., 2010). In Chinese bayberry, anthocyanins are responsible for the red coloration in the presence of light, and bagging of the fruits has been shown to reduce anthocyanin amount to 0.5% of that of non-bagged fruits (Niu et al., 2010).

Light has also been recognized as an important regulator of flavonoid accumulation in species of Rosaceae family such as strawberry (*Fragaria* × *ananassa*, Anttonen et al., 2006; Kadomura-Ishikawa et al., 2013), peach/nectarine (*Prunus persica*, Jia et al., 2005; Ravaglia et al., 2013), pear (*Pyrus pyrifolia*, Feng et al., 2010; Sun et al., 2014), and apple (*Malus* × *domestica*, Takos et al., 2006a,b; Feng et al., 2013). In the apple skin, sunlight is the most important environmental factor inducing flavonoid biosynthesis, especially anthocyanin and flavonol biosynthesis, and fruits with sun-exposed peel have higher levels of anthocyanins and flavonols than those grown in shade (Feng et al., 2013; Li et al., 2013). Exposure of shaded fruit skin to sunlight has been demonstrated to lead up-regulation of flavonol biosynthetic gene *MdFLS* and several anthocyanin biosynthetic genes, including *MdCHS, MdCHI, MdF3H, MdDFR1*, leucoanthocyanidin dioxygenase (*MdLDOX*) and *MdUFGT* (Feng et al., 2013; Vimolmangkang et al., 2014). The elevated expression of flavonoid biosynthetic genes was accompanied by increased levels of anthocyanins, flavonols, and total phenolics (Feng et al., 2013). It has been shown that exposure of bagged apple fruits to light can cause increase in *MdCHI* transcription level by even up to 240-fold, accompanied by *MdCHS* and *MdLDOX* at 80- and 60-fold, respectively, (Takos et al., 2006b). In fact, in many apple cultivars light exposure is required to stimulate anthocyanin biosynthesis and desirable red skin coloration (Takos et al., 2006a; Feng et al., 2013) and also post-harvest light treatment has been shown to have highly positive influence on apple peel anthocyanin and flavonol contents (Hagen et al., 2007).

The rapid induction of flavonoid biosynthesis that is generally observed under high light conditions reflects the important role of flavonoids in photoprotection. However, in some fruit species flavonoid biosynthesis is less affected by light. In tropical mangosteen (*Garcinia mangostana*) fruit, anthocyanin accumulation is unaffected by light (Palapol et al., 2009) and high light can even decrease anthocyanin biosynthesis in pears (Zhang et al., 2011). Moreover, all fruits do not require strong light exposure to accumulate high amounts of flavonoids. For example, bilberry is one of the best sources of anthocyanins although prefers shaded growth habitats and thus anthocyanin accumulation is under strong developmental control (Jaakola et al., 2004). In these kinds of fruits, the spatiotemporal regulation directs the biosynthesis of different classes of flavonoids at different stages of fruit development and environmental factors have only fine-tuning affect. It has been shown that anthocyanin levels are affected more by fruit developmental stages whereas flavonols and PAs are more sensitive to environmental factors (Carbone et al., 2009). For example, flavonol biosynthesis can be induced by light exposure at such stages of grape berry development when flavonols are not normally accumulated (Downey et al., 2004; Matus et al., 2009).

Flavonoid biosynthesis also seems to be influenced by light in a cultivar-specific manner. For instance, among grapevines, sunlight induces both anthocyanin and flavonol accumulation in Cabernet Sauvignon (Matus et al., 2009) while only flavonol production is induced in Shiraz grapes (Downey et al., 2004). Different ability to accumulate anthocyanins under light exclusion between two grape cultivars (Jingyan and Jingxiu) was shown to be related to differences in expression of *UFGT* (Zheng et al., 2013). Also in apple, centuries of intensive breeding has provided red and yellow/green cultivars with varying response to light-stimulated anthocyanin biosynthesis (Feng et al., 2013). Cultivars differing in their sensitivity to light in the induction of flavonoid biosynthesis have also been reported in sweet cherry (*Prunus avium*, Kataoka et al., 2005) and plum (*P. salicina*, Murray et al., 2005), tomato (Giuntini et al., 2008) as well as in pear (Zhang et al., 2011; Qian et al., 2013). Many fruit species also have white varieties in which light do not stimulate anthocyanin production, due to mutations in structural or regulatory genes of flavonoid pathway. These kinds of mutations have been reported for example in grape berries (Kobayashi et al., 2004; Walker et al., 2007), strawberry (Salvatierra et al., 2010), Chinese bayberry (Niu et al., 2010), and bilberry (Jaakola et al., 2002, 2010), and these mutants have had an important role when revealing the regulatory genes involved in the flavonoid biosynthetic pathway.

## Regulation of fruit flavonoid biosynthesis by light

During recent years our knowledge on regulation of flavonoid biosynthesis in fruits has significantly increased through identification of the key transcription factors and genome sequencing of important flavonoid accumulating fruit crops such as grapevine, apple, peach and strawberry (Jaillon et al., 2007; Velasco et al., 2010; Shulaev et al., 2011; Verde et al., 2013). These studies indicate that the R2R3 MYB transcription factors, which directly affect the expression of the structural flavonoid biosynthesis genes, are the primary regulators of fruit flavonoid biosynthesis and have been recently reviewed also in the case of fruit bearing species (Allan et al., 2008; Petroni and Tonelli, 2011; Czemmel et al., 2012; Jaakola, 2013).

The R2R3 MYB transcription factors coordinately regulate flavonoid structural genes by activating or repressing their expression. Recently, R2R3 MYB transcription factors associated with flavonoid biosynthesis have been identified and characterized in several fruit producing species and some of them have been found to respond to light. Light-inducible R2R3 MYB transcription factors controlling flavonoid biosynthesis in fruits have been identified in apple, pear, nectarine, Chinese bayberry, strawberry, litchi, and grapevine (Table 1). In changing light conditions, the expression level of these R2R3 MYB transcription factors is adjusted to regulate the biosynthesis of distinct flavonoid compounds.

**Table 1:** **Identified light-inducible R2R3 MYB transcription factors regulating flavonoid biosynthesis in fruit producing species**.

**Species**	**R2R3 MYB**	**Function**	**References**
Apple (*Malus* × *domestica*)	MdMYB1	Anthocyanin biosynthesis in fruit skin	Takos et al., 2006a
	MdMYBA	Anthocyanin biosynthesis in fruit skin	Ban et al., 2007
	MdMYB10	Anthocyanin biosynthesis in fruit skin	Feng et al., 2013
	MdMYB9	Proanthocyanidin biosynthesis in leaves	Gesell et al., 2014
	MdMYB11	Proanthocyanidin biosynthesis in leaves	Gesell et al., 2014
Chinese bayberry (*Myrica rubra*)	MrMYB1	Anthocyanin biosynthesis in fruit	Niu et al., 2010
Grape (*Vitis vinifera, Vitis* × *labruscana*)	VvMYBF1	Flavonol biosynthesis in fruit skin	Czemmel et al., 2009; Azuma et al., 2012
	VvMYB12	Flavonol biosynthesis in fruit skin	Matus et al., 2010; Liu et al., 2014
	VvMYBA1	Anthocyanin biosynthesis in fruit skin	Jeong et al., 2004; Matus et al., 2009; Azuma et al., 2012; Koyama et al., 2012
	VlMYBA2	Anthocyanin biosynthesis in fruit skin	Azuma et al., 2012
	VvMYBPA1	Proanthocyanidin biosynthesis in fruit skin	Azuma et al., 2012; Koyama et al., 2012
	VvMYBPA2	Proanthocyanidin biosynthesis in fruit skin	Koyama et al., 2012
	VvMYB5a	General flavonoid biosynthesis in fruit skin	Matus et al., 2009; Koyama et al., 2012
	VlMYB5b	General flavonoid biosynthesis in fruit skin	Azuma et al., 2012
Litchi (*Litchi chinensis*)	LcMYB1	Anthocyanin biosynthesis in fruit pericarp	Lai et al., 2014
Nectarine (*Prunus persica*)	PpMYB10	Anthocyanin biosynthesis in fruit skin	Ravaglia et al., 2013
Pear (*Pyrus pyrifolia*)	PyMYB10	Anthocyanin biosynthesis in fruit skin	Feng et al., 2010; Zhang et al., 2011
Cultivated strawberry (*Fragaria* × *ananassa*)	FaMYB10	Anthocyanin biosynthesis in fruit	Miyawaki et al., 2012
Woodland strawberry (*Fragaria vesca*)	FvMYB10	Anthocyanin biosynthesis in flower petal	Lin-Wang et al., 2010

In grapevine, intensive studies have led to identification of multiple R2R3 MYB transcription factors and at the same time shown that the regulation of flavonoid biosynthesis through these factors forms a complex network (reviewed in Czemmel et al., 2012). Various R2R3 MYB transcription factors of grapevine can regulate same branch in flavonoid biosynthetic route but one transcription factor can also regulate both early and late biosynthetic genes and be affected by cues from both developmental and environmental signals (Czemmel et al., 2012; Lai et al., 2013). In grapevine, light has been demonstrated to induce expression of an array of R2R3 MYB transcription factors that are positive regulators of general flavonoid pathway (*VvMYB5a*) as well as those specifically responsible for anthocyanin (*VvMYBA1, VvMYBA2*), flavonol (*VvMYBF1, VvMYB12*), and PA (*VvMYBPA1, VvMYBPA2*) biosynthesis (Table 1). Contradictory results of light-regulation of *VvMYB5b* have been reported (Matus et al., 2009; Azuma et al., 2012; Koyama et al., 2012). However, *VvMYB4*, a repressor of anthocyanin biosynthesis, has not been found to respond light (Matus et al., 2009; Azuma et al., 2012). In fact, none of the so far identified R2R3 MYB transcription factors that have role as a repressor of flavonoid biosynthesis in fruits have been reported to respond to light. For example in flower petals of woodland strawberry (*Fragaria vesca*), high light induced expression of positive regulator *FvMYB10* but not *FvMYB1*, a repressor of anthocyanin biosynthesis (Lin-Wang et al., 2010).

Most of the identified R2R3 MYB transcription factors of flavonoid biosynthesis seem to interact with bHLH and WD40-repeat proteins to form MBW regulatory complex. The role of bHLH and WD40 partners in light-regulated flavonoid biosynthesis is not yet clear. Matus et al. (2009) did not found expression of either MYCA1 or WDR1 to change by influence of light exposure in grapevine, although these genes seem to have a role in other types of stresses (Matus et al., 2010). Zhang et al. (2011) got contradictory results from the expression of bHLH and WD40 partners between two pear cultivars exposed to light. It has earlier been proposed that the MYB partner of the MBW complex would be more directly involved in the light-mediated regulation of flavonoid biosynthesis than bHLH or WD40 partners that would mostly have a co-operative role in the process (Hartmann et al., 2005; Matus et al., 2009, 2010). Recently, bHLH3 but not WD40 of the regulators of flavonoid biosynthesis in nectarine, was shown to be up-regulated after light treatment (Ravaglia et al., 2013), and light-inducible bHLH was suggested as regulator of anthocyanin biosynthesis in apple fruit exocarp (Vimolmangkang et al., 2014).

R2R3 MYB transcription factors mediate direct and specific interaction with MYB recognition element (MRE) that is present in the promoters of the structural target flavonoid genes. The MRE has been found to be necessary for light-induced expression of the structural flavonoid genes, such as CHS (Feldbrügge et al., 1997). Hartmann et al. (2005) showed that the MRE present in promoter of *Arabidopsis* CHS is part of the light regulatory unit (LRU) that is needed for light-mediated induction of CHS. Since then, LRUs as well as other light-responsive elements have been reported not only in promoters of flavonoid structural genes, such as *MdDFR* and *MdUFGT* in apple (Takos et al., 2006a), *VvFLS1* in grapevine (Czemmel et al., 2009), and many structural anthocyanin biosynthetic genes of peach (Zhou et al., 2013) but also promoters of R2R3 MYB transcription factors of grapevine *VvMYBF1*, litchi *LcMYB1* (Lai et al., 2014) and pear *PyMYB10* (Feng et al., 2010). In addition that the presence of LRU in the promoter is an indicator of the light responsiveness of the gene, it also contains bZIP recognition element (ACE). Today, the signaling pathway from light perception to flavonoid biosynthesis through R2R3 MYB transcription factors to induce biosynthesis of specific flavonoid compounds in fruits is not well understood. However, it has been proposed that bZIP transcription factors have a role in this process (Hichri et al., 2011). The involvement of bZIP transcription factor regulating the expression of R2R3 MYB transcription factor attending to flavonoid biosynthesis in fruit was demonstrated recently for the first time when bZIP transcription factor was shown to regulate PA biosynthesis in persimmon (*Diospyros kaki*) fruit (Akagi et al., 2012).

**Figure 3:**
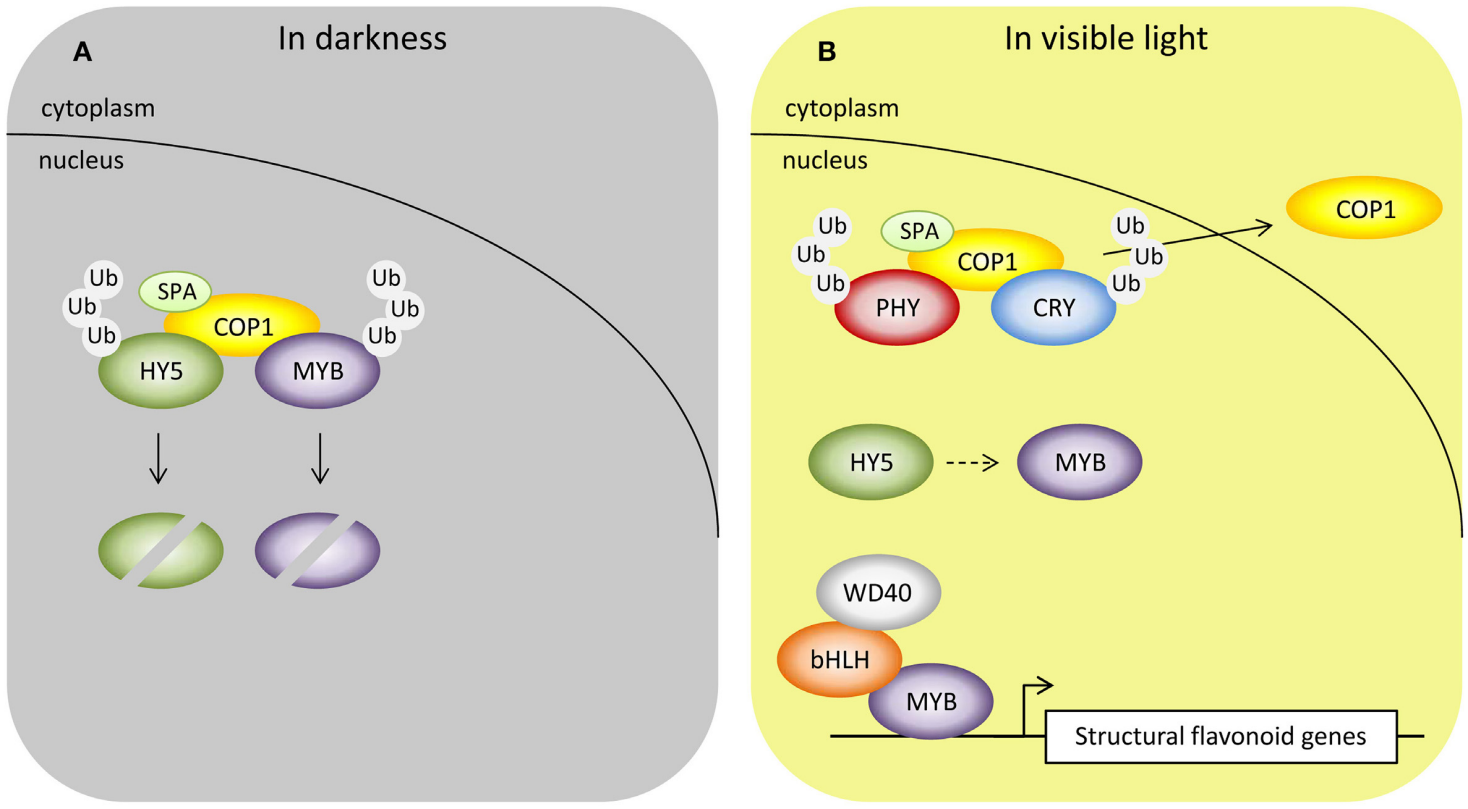
**COP1 acts as a central repressor in light signaling pathway by interacting directly with photoreceptors to mediate different light-regulated plant developmental processes. (A)** In darkness, nuclear localized COP1 targets positive regulators, such as transcription factors HY5 and R2R3 MYBs, for ubiquitination and subsequent protein degradation through a 26S proteasome pathway. **(B)** In visible light, interaction with activated photoreceptors repress function of COP1 that is subsequently exported from nucleus allowing nuclear-localized transcription factors to accumulate and induce gene expression in light-regulated processes. The expression of structural flavonoid genes is directly regulated by R2R3 MYB transcription factors which may be regulated by bZIP transcription factor such as HY5. During the process, photoreceptors are ubiquitinated by COP1 and targeted for degradation (Lau and Deng, 2012).

Recently, an important piece of the puzzle in the mechanism by which light controls anthocyanin biosynthesis in fruits at post-translational level was reported. Li et al. (2012) demonstrated that in apple, the MdMYB1 protein, a positive regulator of light-induced anthocyanin biosynthesis, interacts directly with MdCOP1 protein that negatively modulates abundance of the MdMYB1. The RING-finger type ubiquitin E3 ligase CONSTITUTIVE PHOTOMORPHOGENIC1 (COP1) acts as a negative regulator of light signaling directly at the downstream of the photoreceptors and control different light-regulated plant development processes by adjustment of its subcellular localization (Lau and Deng, 2012). The physical protein-protein interaction of COP1 with phytochromes, cryptochomes and phototropins has been demonstrated in *Arabidopsis* (Jang et al., 2010; Jeong et al., 2010; Liu et al., 2011). In the dark, COP1/SUPPRESSOR OF PHYA (SPA) complex is localized in the nucleus, where it interacts with the subset of specific key positive regulators mediating their ubiquitination and degradation via the 26S proteasome pathway (Figure 3A). Ubiquitination involves covalent attachment of ubiquitin polypeptides to target protein, which are subsequently marked for degradation by 26S proteasome that mediates proteolysis of target protein (Vierstra, 2009). In light, COP1/SPA complex interacts with activated photoreceptors leading to the inhibition of COP1/SPA function by dissociation of COP1 from the complex and exportation from the nucleus (Figure 3B). Low abundance of COP1 in nucleus allows nuclear-localized transcription factors to accumulate and induce gene expression (Lau and Deng, 2012).

Among COP1 targeted transcription factors, ELONGATED HYPOCOTYL5 (HY5), a bZIP transcription factor promoting photomorphogenesis (Lee et al., 2007), is a direct target of COP1. HY5 becomes stabilized in light when the COP1 protein is removed from nucleus (Figure 3B). HY5 has also been linked to activation of the R2R3 MYBs and key structural genes of the flavonoid pathway as well as the accumulation of flavonoids in response to light in *Arabidopsis* and apple (Hardtke et al., 2000; Stracke et al., 2010; Maier et al., 2013; Peng et al., 2013; Shin et al., 2013). However, other yet unidentified more rapid mechanism mediating COP1 signal from phytochomes and cryptochromes have been proposed since export of COP1 from nucleus is rather slow (24 h) and thus allows transcription factors to accumulate only at extended light conditions (Lau and Deng, 2012). Yet accumulation of HY5 and light-induced expression of flavonoid pathway genes can be seen rapidly within few hours (Cominelli et al., 2008; Li et al., 2010).

Mutations in COP1/SPA complex has been shown to lead in increased accumulation of anthocyanins in *Arabidopsis* (Maier et al., 2013) and mutation in light signaling machinery has been shown to have effects on flavonoid biosynthesis also in fruits. Tomato *hp* mutants, characterized by exaggerated light responsiveness, overproduce several flavonoid compounds in mature fruits (Azari et al., 2010). Furthermore, additional mutation in ANTHOCYANIN FRUIT (*AFT*), gene encoding MYB transcription factor, in tomato *hp* mutant positively affects fruit flavonoid content (Azari et al., 2010). Also inactivation of DE-ETIOLATED1 (DET1), a photomorphogenesis regulating gene, has been shown to lead to a significant increase in flavonoid content in tomato fruit (Davuluri et al., 2005).

The regulation of anthocyanin biosynthesis has recently been suggested to be closely linked with other protective processes against high light. Shading experiments with peach leaves by Zhou et al. (2013) demonstrated simultaneous reduction in anthocyanin biosynthesis with the increase in expression of photorespiratory genes. Also high expression level of photorespiratory genes in *Arabidopsis* CHI/F3′H mutant, that are unable to accumulate anthocyanins, suggest that photorespiration-related genes may be involved in the regulation of anthocyanin biosynthesis but the mechanism is so far unknown (Zhou et al., 2013).

## Effect of light quality on flavonoid biosynthesis in fruits

### Visible light wavelengths

The accumulation of fruit flavonoids is also sensitive to the quality of the light spectrum received by the plant. It appears that shorter wavelengths, in the range of blue and UV-light show the most prominent effect in the accumulation of flavonoids in fruits, often by increasing the expression of flavonoid pathway genes. In unripe strawberries, blue light increased significantly the biosynthesis of anthocyanins and the expression of *FaCHS* after four days of treatment (Kadomura-Ishikawa et al., 2013). In grape berries treated with light emitting diode (LED) light, anthocyanin concentrations were highest in blue light-treated skin, followed by red light treatment (Kondo et al., 2014). In the same study, differences in anthocyanin profile were also detected and especially malvidin-glycosides increased toward harvest in blue and red LED-treated skin, unlike in untreated controls. In this case however, the transcript levels of *VlMYBA1-2, VlMYBA2*, and *VvUFGT* did not necessarily coincide with anthocyanin concentrations. These findings have increased the interest toward the application of led lights in fruit orchards in order to improve fruit quality at ripeness in terms of nutritional value and content of bioactive compounds.

The relevance of blue light perception through phototropins in flavonoid biosynthesis has recently been demonstrated at molecular level in strawberry, when expression of phototropin 2, *FaPHOT2*, was shown to elevate during berry development and correspond to increase in anthocyanin content (Kadomura-Ishikawa et al., 2013). Furthermore, in the same study knockdown of *FaPHOT2* resulted in decreased anthocyanin content while overexpression increased accumulation of anthocyanins in strawberry fruit. Also, the overexpression of blue light sensing cryptochrome in tomato (Giliberto et al., 2005) resulted in the accumulation of anthocyanins in tomato fruits.

Interestingly, the accumulation of flavonoid compounds in response to light has been shown to continue in post-harvest fruits. Total anthocyanin content of strawberry fruits significantly increased after four days of treatment with blue light (40 μmol m^−2^ s^−1^) at 5°C compared to the control fruits (Xu et al., 2014). Meanwhile, the treatment also increased the activities of the enzymes of the general phenylpropanoid and flavonoid pathway including glucose-6 phosphatase (G6PC) phenylalanine ammonialyase (PAL), cinnamate-4-hydroxylase (C4H), 4-coumarate: coenzyme A ligase (4CL), CHS, F3H, DFR, ANS, and UFGT. Therefore, a supplemental blue light source might increase anthocyanin content in strawberries also during fruit storage. These results are important as they indicate that the physiological response to light stimulus is located in the fruit.

### UV-light

Solar UV radiation reaching the earth's surface is composed of UV-A (320–400 nm) and part of UV-B (280–320 nm) while most of the UV-B and all UV-C (<280 nm) radiation is absorbed by the ozone layer. Over the last decades, depletion in ozone layer has increased the level of solar UV-B radiation reaching the earth and now approximately 0.5% of total solar light radiation accounts from UV-B (Heijde and Ulm, 2012). Although UV-B represents only a small fraction of total solar spectrum, it has extensive photobiological effects on plants inducing changes in photosynthesis, cell division and other life processes that affect growth and development of plants (Jansen et al., 1998; Cockell and Knowland, 1999; Sullivan and Rozema, 1999; Hollósy, 2002; Kakani et al., 2003; Brown et al., 2005; Mpoloka, 2008).

Stress caused by UV-B light is known to enhance the production of reactive oxygen species (ROS) damaging DNA, proteins and photosynthetic apparatus in plants, but these effects are dose and phenotype dependent (Smith et al., 2000; Frohnmeyer and Staiger, 2003). Some flavonoids, especially flavonols, are reported to be highly effective scavengers of ROS as well as selectively absorbing UV-B radiation (Falcone Ferreyra et al., 2012). Therefore, it is not surprising that flavonoid production in plants is strongly induced by light and UV-B wavelengths. Many flowers and fruits produce flavonols, flavones, and anthocyanins as response to excess UV-light. However, the mechanisms involved in the induction of these metabolites need to be discussed in terms of the resistance and acclimation of plants.

The effects of UV-light in plants have been studied actively since late 1970s' when the depletion in the ozone layer was discovered in the polar regions (Farman et al., 1985). Still, the future of the earth's UV climate is uncertain (Andrady et al., 2010) emphasizing the importance of the topic especially for agriculture affecting the yield and quality of crop plants including fruits. In addition to UV-light effects under natural growth conditions, post-harvest treatments with UV-light have been performed to improve fruit quality. Most of these studies have been carried out with UV-C radiation, which is more energetic and can rapidly decrease the incidence of pathogens in fruits (Stevens et al., 1996; Allende and Artés, 2003). UV-B radiation has been applied post-harvest with the special purpose to increase the contents of health beneficial secondary metabolites.

#### Natural UV-light

The effect of natural UV-B radiation on the biosynthesis of flavonoids was studied in an experiment where Cabernet Sauvignon grape berries were cultivated under UV-shield (Koyama et al., 2012). A high decrease in the content of flavonols was detected in the skin of grape berries (under UV-shield), whereas levels of PAs and cinnamic acids were less affected. Additional experiment under artificial white light compared with white light together with supplemental UV-light revealed that UV-light did not markedly affect the levels of PAs in grape berry skin. The transcript levels of analyzed flavonoid pathway genes were consistent with metabolite results and a decrease in *VvFLS4* transcript abundance was detected in berry skin samples under UV-shield (Koyama et al., 2012).

Liu et al. (2014) reported the effect of natural UV-B radiation related biosynthesis on flavonoid biosynthesis in white Sauvignon blanc grape berries. The experiment was conducted in New Zealand, where the natural UV radiation levels are high compared with corresponding latitudes in Northern hemisphere, using A-frame-mounted UV-transmitting/excluding screens covering only the fruiting zone of the plants. Substantial increase in the levels of flavonols, particularly quercetin and kaempferol glycosides was detected upon fruit exposure to UV-B (Table 2). Of five *VvFLS* genes of grapevines, two were found to be transcriptionally active, and only one (*VvFLS4*) was responsive to UV-B. Of the related transcription factors (*VvMYB12, VvMYCA1, VvWDRs*), only *VvMYB12* was found to be responsive to UV-B. In the same study, other candidate genes associated with low and high UV-B fluence responses (*VvUVR8, VvHY5, VvCOP1, VvCHS*) showed variable results.

**Table 2:** **Main responses of flavonoid compounds under UV-light exposition in fruits**.

**Species**	**Tissue**	**UV type and experimental conditions**	**Metabolites**	**Response**	**References**
Apple (*Malus domestica*) cv. Braeburn^a^/Granny Smith^b^	Skin	Post-harvest UV-B	Quercetin-3-O-glycoside	a / b	Solovchenko and Schmitz-Eiberger, 2003
		97.0 kJ m^−2^		+ n	
		2.5 h			
cv. Aroma	Peel	Post-harvest UV-B shade grown/sun exposed	Epicatechin	+/+	Hagen et al., 2007
			Procyanidin (B1+B2)	n/n	
			Phloridzin	n/n	
			Quercetins (galactoside, glucoside, and rhamnoside)	+/n	
			Cyanidin-3-galactoside	+/+	
Blueberry (*Vaccinium corymbosum*) cv. Duke	Complete fruit	Post-harvest UV-C	Myricetin-3-O-arabinoside	–	Wang et al., 2009b
		4.3 kJ m^−2^	Quercetin-3-O-galactoside	–	
		24 h ^(high dose)^	Quercetin-3-O-glucoside	–	
			Kaempferol-3-O-glucoside	n	
			Kaempferol-3-O-glucuronide	–	
			Delphinidin-3-O-galactoside	–	
			Dephinidin-3-O-arabinoside	–	
			Cyanidin-3-galactoside	n	
			Petunidin-3-O-galactoside	–	
			Petunidin-3-O-glucoside	–	
			Petunidin-3-arabinoside	–	
			Malvidin-3-O-galactoside	–	
			Malvidin-3-O-arabinoside	–	
*Grapevine (Vitis vinífera*) cv. Cabernet Sauvignon	Skin	Post-harvest	2,3 -*cis*- flavan-3-ols	+/–/+	Zhang et al., 2013
		UV-A/B/C	2,3- *trans*-flavan-3-ols	+/–/+	
		1.8 kJ m^−2^	(-)-epigallocatequin	+/–/+	
		3-week old berries^*^	[(-)-epigallocatequin-3-O-gallate + (-)-epicatequin + catechin]	+/+/+	
		7-week^*^		n/+/+	
				+/+/+	
				+/+/+	
				n/+/+	
		11-week^*^		–/–/–	
				–/–/–	
				–/n/–	
				–/–/–	
cv. Sauvignon blanc	Skin	Solar UV-B	Quercetin-3-O-glucoside	+	Gregan et al., 2012; Liu et al., 2014
		6 weeks post-veraison	Quercetin-3-O-glucuronide	+	
			Quercetin-3-O-rutinoside	+	
			Isorhamnetin-3-O-glucoside	+	
			Kaempferol-3-O-glucoside	+	
			Kaempferol-3-O-rutinoside	+	
			Kaempferol-3-O-glucuronide	+	
cv. Tempanillo	Skin	Supplemental UV-B	Kaempferol-3-O-galactoside	+/+	Martínez-Lüscher et al., 2014
		9.66 kJ m^−2^ d^−1^	Kaempferol-3-O-glucoside	+/+	
		set to ripness/onset of veraison to ripness	Quercetin-3-O-galactoside	+/+	
			Quercetin-3-O-glucuronide	+/+	
			Quercetin-3-O-glucoside	+/+	
			Isorhamnetin-3-O-glucoside	n/n	
			Syringetin-3-O-glucoside	n/n	
			Delphinidin-3-O-glucoside	n/n	
			Cyanidin-3-O-glucoside	n/n	
			Petunidin-3-O-glucoside	+/n	
			Peonidin-3-O-glucoside	+/n	
			Malvidin-3-O-glucoside	n/n	
Peaches and nectarine *(Prunus persica)* cv. Suncrest^a^/Babygold^b^/Babygold^c^	Skin	Post-harvest UV-B	Quercetin-3-O-diglucoside	a / b / c	Scattino et al., 2014
		146 kJ m^−2^	Quercetin-3-O-galactoside	+ − −	
		24 h	Quercetin-3-O-rutinoside	+ n n	
			Quercetin-3-O-glucoside	+ n n	
			Kaempferol-3-O-galactoside	+ − −	
			Kaempferol-3-O-rutinoside	+ n n	
			Kaempferol-3-O-glucoside	n + +	
			Isorhamnetin-3-O-galactoside	+ n n	
			Isorhamnetin-3-O-rutinoside	+ n n	
			Isorhamnetin-3-O-glucoside	− n n	
			Cyanidin-3-O-glucoside	− n n	
				+ + nd	
Tomato (*Solanum lycopersicum*) cv. Money Make^a^/*hp-1* mutant^b^	Skin	Post-harvest UV-B	Naringenin	a / b	Castagna et al., 2014
		6.08 kJ m^−2^d^−1^	3-quercetin-pentosyl-rutinoside		
		mature green fruit stage	Rutin (quercetin 3-O-rutinoside)		
	Flesh			+ +	
				+ n	
				+ n	
				nd nd	
				n n	
				n +	

+, significantly induced; −, significantly reduced; n, no variation compared to control; nd, not detected; a/b/c, different genotype from the same species.

* Main responses between hours (Zhang et al., 2013).

**Figure 4:**
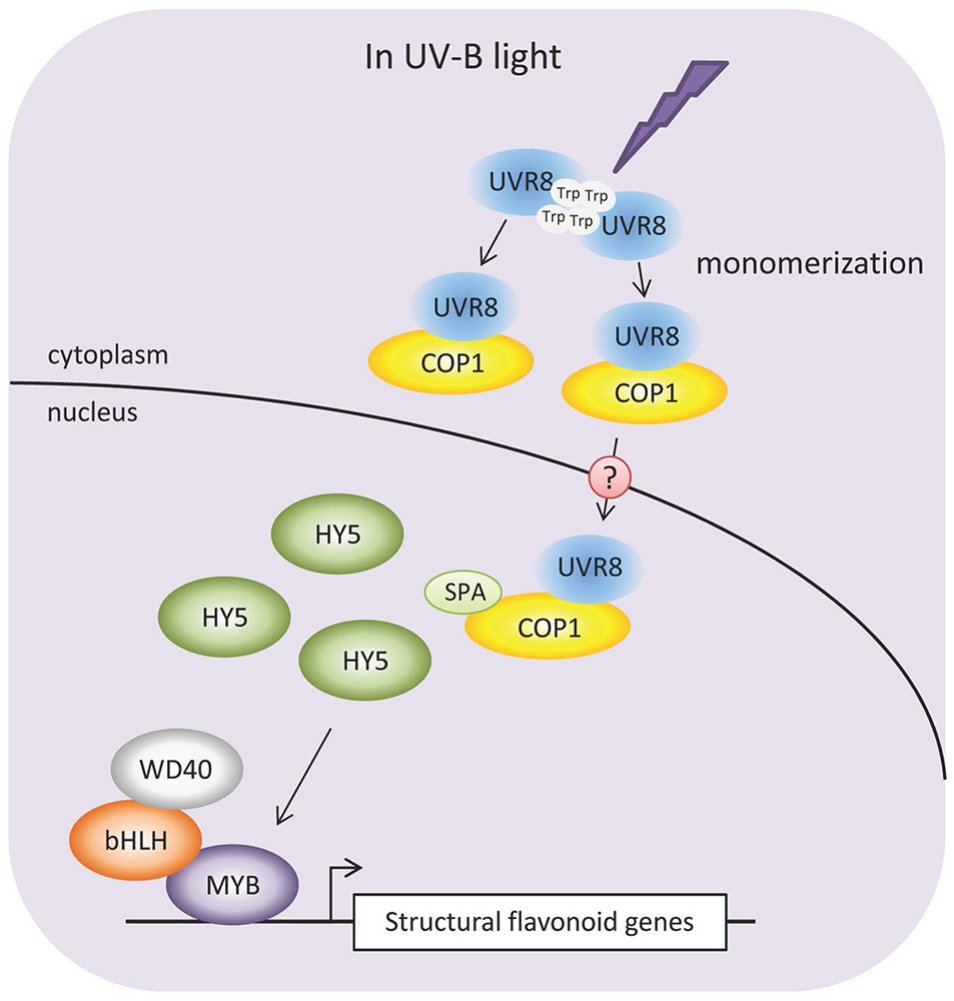
**Proposed mechanism for signaling pathway affecting flavonoid biosynthesis under UV-B radiation**. UV-B radiation is strongly absorbed by tryptophan (Trp) amino acid residues in the dimeric form of UVR8 photoreceptor leading to the monomerization of UVR8. Monomeric UVR8 and COP1 form a complex that accumulates in the nucleus of the cells. The UVR8-COP1-SPA complex stabilizes bZIP transcription factor HY5 promoting the activity of different R2R3 MYBs for the transcription of specific flavonoid biosynthesis genes (Favory et al., 2009; Christie et al., 2012; Jenkins, 2014; Li et al., 2014).

Similarly to other photoreceptors, signaling of the UV-B absorbing photoreceptor UVR8 is also mediated through COP1. Contrary to the role of COP1 in visible light, under UV-B irradiation *Arabidopsis* COP1 has been shown to act as a positive regulator by interacting with UVR8 and promote through an unknown mechanism the expression of HY5 (Lau and Deng, 2012; Figure 4). In the process, UVR8 changes from a dimer to monomer to interact with COP1. The recent results have given evidence on the function of the same mechanism mediating the responses of the UV-B radiation also in apple (Peng et al., 2013). In grape berries, *VvUVR8* did not respond to UV-B but instead showed fruit development related changes having significantly higher expression at pre-veraison compared to post-veraison (Liu et al., 2014). This result is consistent with previous findings in *Arabidopsis* showing that UVR8 does not respond to different light qualities (Kaiserli and Jenkins, 2007). In contrast, *VvHY5* did show a significant up-regulation by UV-B light as well as *VvCHS1* and *VvCHS2*. The results suggested that flavonol biosynthesis in grape is stimulated predominantly through the low fluence UV-B response pathway (Liu et al., 2014). It is notable that more than 98% of the UV-light reaching the earth's surface correspond to UV-A radiation. There has been suggestions of the existence of specific photoreceptor for UV-A, different than UVR8, but it still remains uncertain (Zhou et al., 2007; Guo and Wang, 2010; Wang et al., 2012).

#### Pre- and post-harvest studies with supplemental UV-light

The effect of supplemental UV-light treatments on the content of phenolic compounds has been evaluated in various fruit crops, often with the aim of developing techniques to increase health-promoting potential of the fruits and indirectly improve the aesthetic value with higher anthocyanin content. The changes in the content of phenolic compounds in response to UV-light vary between species. Early pre-harvest studies showed the enhancement of anthocyanin levels as response to UV irradiation in apple (Arakawa et al., 1985) and sweet cherry skin (Arakawa, 1993; Kataoka et al., 1996). More recently, UV-B irradiation treatment has been shown to increase the anthocyanin content and the expression of *MdMYBA* and anthocyanin pathway genes in apple skin (Ban et al., 2007; Peng et al., 2013).

Martínez-Lüscher et al. (2014) exposed red grapevine variety (cv. Tempranillo) to two doses (5.98 and 9.66 kJ m^−2^ d^−1^) of supplemental UV-B radiation, under controlled conditions, in order to study the effect on grape traits including flavonoid profile. The contents of anthocyanins and flavonols were enhanced by UV-B in grape berry skin, and qualitative differences were also detected in the flavonol profiles compared to untreated fruits (Martínez-Lüscher et al., 2014; Table 2). In Cabernet Sauvignon grapevine variety, the accumulation of flavan-3-ols was found to be developmental stage-dependent in response to the three types of supplemental UV-light (Zhang et al., 2013). Supplemental UV-A irradiation promoted flavan-3-ol accumulation and transcript levels of related genes at all studied early developmental stages (3–11 weeks after flowering), whereas UV-B and UV-C were effective only grape berries of 7–11 weeks after flowering. The results indicated that UV radiation increased flavan-3-ol levels during the berry development but did not increase the flavan-3-ol content in the mature berries (Zhang et al., 2013).

Significant post-harvest effects of UV-B exposure on flavonoid biosynthesis has been reported in different nectarine varieties. The accumulation of anthocyanins in the skin of nectarines (cv. Stark Red Gold) exposed to white light supplemented with UV-light was after 72 h in accordance with enhanced transcript levels of flavonoid pathway genes such as *PpDFR* and *PpUFGT* (Ravaglia et al., 2013). Particularly, the *PpMYB10* gene was strongly responsive to the treatment but the levels of PAs and flavonols were not changed during the experiment. However, increment in the transcript level of *PpFLS1* was reported, which suggests the accumulation of flavonols in a longer period than 72 h (Ravaglia et al., 2013). In apple, the exposure of post-harvest fruits to visible light supplemented with UV-B also increased the content of total flavonoids, and in particular quercetin-glycosides and anthocyanins, in shade-grown fruits of cultivar Aroma (Hagen et al., 2007). Another post-harvest treatment with UV-B/visible light showed similar results in European pear (*P. communis*) and Chinese sand pear (*P. pyrifolia* Nakai, Qian et al., 2013; Sun et al., 2014). The UV-B radiation also increased significantly flavonoid levels, especially flavonols, in the flesh of post-harvest tomato fruits at green mature stage (Castagna et al., 2014), whereas UV-A caused significant short term increment in anthocyanin content in tomato fruits at green mature stage (Guo and Wang, 2010).

Genotype-related differences were detected in a study focusing also on post-harvest effects of UV-B irradiation, in which fruits of cv. Suncrest and cv. Babygold 7 of peach and cv. Big Top of nectarine were irradiated with UV-B. Fruits of cultivars Big Top and cv. Suncrest responded by increasing the levels of flavonol and anthocyanidin glycosides whereas in cv. Babygold 7 that is lacking anthocyanins, flavonol levels decreased after UV-B irradiation (Table 2). The transcript levels of the structural phenylpropanoid and flavonoid pathway genes (*PpC4H, Pp4CL, PpF3H, PpDFR, PpCHI, PpPAL, PpCHS, PpLDOX*) were consistent with the detected metabolite levels (Scattino et al., 2014).

The post-harvest treatments with UV-C light have been reported to delay fruit senescence and increase the antioxidant activity and flavonoid content in fruits. Wang et al. (2009b) tested the effect of UV-C dosages from 0.43 to 6.45 kJ m^−2^ (1–15 min treatments) on flavonoid contents and antioxidant activity in post-harvest blueberries (*V. corymbosum*). The results indicated substantial increase in flavonol and anthocyanidin glycosides and antioxidant activities instantly after the treatments. However, the contents decreased to the same level with the untreated control berries after 19 h from the treatments. In strawberry, the effect of 4.1 kJ m^−2^ UV-C radiation on anthocyanin content, antioxidant activity, and overall quality was studied in post-harvest berries at the large green maturity stage (Li et al., 2014). UV-C induced accumulation of anthocyanins and flavonols as well as activity of the enzymes of the phenylpropanoid pathway, but after 3 days under UV-C light these effects were not detected anymore. UV-C irradiation also positively enhanced the content of stilbene cis- and trans-piceid together with quercetin-3-*O*-galactoside and quercetin-3-*O*-glucoside in grape berry skin up to 3-fold respect to control grape berries (Crupi et al., 2013). UV-C irradiation has also been shown to increase the radical scavenging properties of papaya fruit skin due enhanced flavonoid content (Rivera-Pastrana et al., 2013).

## Photoperiod

Photoperiod influences various ways in the growth and development of plants. The photoperiodic conditions can also affect the biosynthesis of secondary metabolites. For instance, the biosynthesis of anthocyanins in *Xanthium* flowers has been shown to be under photoperiodic regulation (Taylor, 1965). Plants possess an internal timekeeping system, circadian clock, which runs on a period of about 24 h. In addition to light perception, circadian clock activity is essential for the detection of photoperiod and subsequent mediation of responses to daily changes in light conditions. Light signaling pathways and circadian clock are interconnected as photoreceptors are involved in entrainment of the clock and the circadian clock in the regulation of the photoreceptor genes (Harmer, 2009; Lopez et al., 2012).

Increased levels of flavonoids, especially anthocyanins under longer photoperiod have been detected in different plant species (Camm et al., 1993; Reyes et al., 2004; Carvalho et al., 2010; reviewed by Jaakola and Hohtola, 2010) but opposite results have also been reported (Steindal et al., 2013). Studies focusing purely on the effect of circadian rhythms on the flavonoid content in fruits are scarce and the results are often difficult to analyze because of many variables. In field studies, along with day length and total irradiation level, diurnal temperature changes also affect the flavonoid biosynthesis. In controlled experiment with bilberry, 24 h day length yielded significantly higher levels of anthocyanins in fruits compared with 12 h day length treatment (Uleberg et al., 2012). Field experiments have also shown higher anthocyanin levels in *Vaccinium* berries growing under longer day length in the northern latitudes (Lätti et al., 2008, 2010; Åkerström et al., 2010). Long-term field experiments with currants (*Ribes* spp.) showed contradictory results in the flavonoid levels between different latitudes (Zheng et al., 2012; Yang et al., 2013). The content of flavonols in red, white and green currants and anthocyanins in red currant cultivars were notably higher in the northern growth habitants whereas opposite was detected in black currant (*R. nigrum*) cultivars. However, these field experiments were performed in long day conditions with the differences in day length varying from about 16 h to 24 h during the growth period.

Increasing evidence has been gathered to support the idea that ubiquitination-mediated targeted protein degradation is involved in controlling day length perception in plants in response to photoperiod. Ubiquitin-mediated protein degradation by COP1 has been shown to be important element in stabilization of circadian clock components in *Arabidopsis* (Lau and Deng, 2012; Piñeiro and Jarillo, 2013).

## Concluding remarks

In conclusion, flavonoid composition in fruits is strongly affected by surrounding light conditions. In general, higher solar radiation tends to increase flavonoid content in fruits. Specific wavelengths can also alter the profile of flavonoids in fruit tissues. However, variation in response can be high between and even within species. Interaction of specific light conditions with other environmental factors can also change the response markedly. Although biosynthesis of flavonoids has been extensively studied, only recently the underlying molecular mechanisms of light-controlled flavonoid biosynthesis have begun to be revealed. Some of the mechanisms shown in model plants, such as COP1-mediated signaling pathways, have recently also been found in fruit tissues. Understanding the regulation of flavonoid biosynthetic pathway as well as involved light signaling machinery in fruit producing species is important to generate and select fruits enriched with flavonoid compounds to obtain desirable dietary and health-beneficial properties. The same compounds can also affect to self-life of the fruits. Moreover, several recent studies show that pre- and post-harvest treatments with selected light conditions have potential for commercial applications.

### Conflict of interest statement

The authors declare that the research was conducted in the absence of any commercial or financial relationships that could be construed as a potential conflict of interest.
